# Proinflammatory and Anabolic Gene Expression Effects of Platelet-Rich Gel Supernatants on Equine Synovial Membrane Explants Challenged with Lipopolysaccharide

**DOI:** 10.1155/2017/6059485

**Published:** 2017-07-06

**Authors:** Jorge U. Carmona, Diana L. Ríos, Catalina López, María E. Álvarez, Jorge E. Pérez

**Affiliations:** ^1^Grupo de Investigación Terapia Regenerativa, Departamento de Salud Animal, Universidad de Caldas, Manizales, Colombia; ^2^Grupo de Investigación Biosalud, Departamento de Ciencias Básicas para la Salud, Universidad de Caldas, Manizales, Colombia

## Abstract

Platelet-rich plasma (PRP) preparations are used in horses with osteoarthritis (OA). However, some controversies remain regarding the ideal concentration of platelets and leukocytes to produce an adequate anti-inflammatory and anabolic response in the synovial membrane. The aims of this study were to study the influence of leukoconcentrated platelet-rich gel (Lc-PRG) and leukoreduced platelet-rich gel (Lr-PRG) supernatants on the quantitative expression of some proinflammatory and anabolic genes in equine synovial membrane explants (SMEs) challenged with lipopolysaccharide (LPS). SMEs from six horses were cultured over 96 h. Then, SMEs were harvested for RNA extraction and quantitative gene expression analysis by RT-qPCR for nuclear factor kappa B (NF*κ*B), matrix metalloproteinase 13 (MMP-13), a disintegrin and metalloproteinase with thrombospondin motifs 4 (ADAMTS-4), collagen type I alpha 1 (COL1A1), collagen type II alpha 1 (COL2A1), and cartilage oligomeric matrix protein (COMP). The 25% and 50% Lc-PRG supernatants led to downregulation of NF*κ*B, MMP-13, ADAMTS-4, COL1A1, COL2A1, and COMP in SMEs. Lr-PRG supernatants (particularly at the 50% concentration) induced downregulation of NF*κ*B, MMP-13, ADAMTS-4, and COL1A1 and upregulation of COL2A1 and COMP. Lr-PRG supernatants should be used for the treatment of inflammatory arthropathies in horses because they have anti-inflammatory and anabolic effects in the synovial membrane.

## 1. Introduction

Currently, platelet-rich plasma (PRP) and other platelet-related products have emerged as a therapeutic option for the treatment of osteoarthritis (OA) in humans [1, 2] and horses [2–6]. However, there are several PRP preparations with different cellular (platelets and white blood cells (WBCs)) and molecular (growth factors (GFs) and cytokines) profiles, which undoubtedly produce different joint tissue responses after they come into contact with these substances [1, 2, 7, 8].

Although not perfected, there is a standard classification for liquid PRP preparations, which comprises two categories: (1) leukoreduced PRP (Lr-PRP) and (2) leukoconcentrated PRP (Lc-PRP) preparations [9]. In a general fashion, Lr-PRP preparations are represented by cell concentrates with variable counts of platelets and without or with negligible numbers of WBCs, whereas Lc-PRP preparations are hemoderivatives with high concentration of leukocytes [9]. Notably, once any PRP preparation is mixed with an activating substance, like calcium salts or thrombin, it is polymerized into a platelet-rich gel (PRG), which is a live biological scaffold with the capacity to retain and release GFs over time [10].

There are controversies about which PRP preparation (either Lr-PRP or Lc-PRP) is better to treat osteoarthritic patients [4, 11, 12]. Some investigators favor the use of Lr-PRP [13], whereas others recommend using Lc-PRP preparations [14]. However, additional research is necessary to address this concern. In line with this, we have performed an in vitro study to evaluate the effects of different concentrations (25 and 50%) of Lr-LPG and Lc-PRG supernatants over a 96 h period on anabolic and proinflammatory gene expression in synovial membrane explants (SMEs) challenged with lipopolysaccharide (LPS).

We evaluated the expression of some key genes (nuclear factor kappa B (NF*κ*B), matrix metalloproteinase 13 (MMP-13), a disintegrin and metalloproteinase with thrombospondin motifs 4 (ADAMTS-4), collagen type I alpha 1 (COL1A1), collagen type II alpha 1 (COL2A1), and cartilage oligomeric matrix protein (COMP)) implicated in joint homeostasis and pathology. Our hypotheses were that both PRG supernatants at different concentrations produce diverse gene expression changes in inflamed SMEs.

## 2. Materials and Methods

This study was approved by the committee on animal experimentation at the authors' institution.

### 2.1. Samples

Synovial membrane samples from the metacarpophalangeal joints from six horses, aged 5 to 11 years, were included in this study. The samples were taken from horses that were free from musculoskeletal disease and euthanized by a pentobarbital intravenous overdose for other medical reasons. All metacarpophalangeal joints were radiographed and macroscopically evaluated to exclude horses with OA-associated joint changes.

### 2.2. Lr-PRP and Lc-PRP Preparation

**Figure 1:**
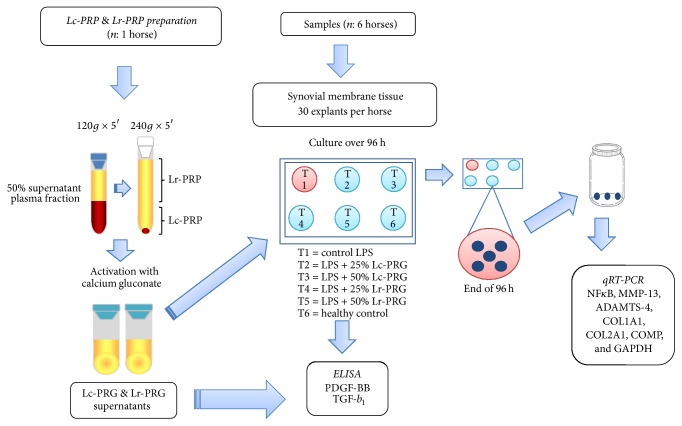
Schematic workflow of the experiments in the study.

Venous blood from one clinically healthy mare was used in order to avoid variability in the GF and cytokine concentrations in the PRG supernatants used in the experiments. Platelet concentrates were obtained through a manual double centrifugation tube method [15]. Blood was drawn from jugular venipuncture and deposited in 4.5 mL tubes with sodium citrate solution (BD Vacutainer®, Becton Drive, Franklin Lakes, NJ, USA). After centrifugation at 120 ×g for 5 minutes, the first 50% of the top supernatant plasma fraction, adjacent to the buffy coat, was collected. This fraction was centrifuged at 240 ×g for 5 minutes, and then the bottom quarter of the fraction was collected [15]. This fraction was designated as Lc-PRP. The upper plasma fraction was designated as Lr-PRP (Figure 1). Whole blood and both PRP preparations were analyzed for platelet and WBC counts using an impedance-based hematology device (Celltac-*α* MEK 6450, Nihon Kohden, Japan).

Both PRP preparations were activated with calcium gluconate (Ropsohn Therapeutics, Ltda., Bogotá, Colombia) (ratio 1 : 10) and incubated at 37°C for 1 h until clot retraction occurred. Fresh Lr-PRG and Lc-PRG supernatants were used to add to the culture media at two concentrations (25% and 50%). Aliquots of both PRG supernatants were frozen at −86°C for later quantification of platelet-derived growth factor BB (PDGF-BB) and transforming growth factor beta 1 (TGF-*β*_1_).

### 2.3. Synovial Membrane Explants Culture and LPS Challenge

Synovial membrane samples were obtained aseptically, and circular 4 mm diameter explants were obtained using a disposable biopsy punch (KAI Medical, Solingen, Germany). SMEs were cultured in culture media standard as described in [12]. After 24 h, SMEs were challenged with 100 ng/mL of LPS (Sigma-Aldrich, St Louis, MO, USA) to induce inflammatory/catabolic damage to these tissues [16].

### 2.4. Study Design

A total of 30 SMEs were obtained from each horse. The study design included the evaluation of six experimental groups using five SMEs per group from each horse as follows: one SME healthy control group without LPS and without the addition of any PRG supernatant, one SME control group challenged with LPS and without the addition of any PRG supernatant, and four SME groups cultured with Lc-PRG and Lr-PRG supernatants at two different concentrations (25% and 50%) and with LPS. After 1 h of incubation, Lc-PRG and Lr-PRG supernatants were added in order to obtain concentrations at 25% and 50%. All SME groups were cultured at 96 h, after which they were deposited in an RNA conserving solution (RNAlater, Life Technologies, Carlsbad, CA, USA) for quantitative gene expression of NF*κ*B, MMP-13, ADAMTS-4, COL1A1, COL2A1, and COMP. A schematic diagram (Figure 1) summarizes the study design and methodology.

### 2.5. ELISA Analysis

Lc-PRG and Lr-PRG supernatants were used to determine the concentration of PDGF-BB and TGF-*β*_1_ by ELISA in duplicate. All proteins were assayed using commercial ELISA kits from R&D Systems (Minneapolis, MN, USA). PDGF-BB (Human PDGF-BB DuoSet, DY220) and TGF-*β*_1_ (Human TGF-*β*_1_ DuoSet, DY240E) were determined using human antibodies due to the high sequence homology between these proteins in humans and horses [17, 18]. Furthermore, these kits have been used for the same purposes in other equine PRP studies [19, 20]. Standards provided for each ELISA kit were used to prepare each standard curve following the manufacturer's instructions. Absorbance readings were performed at 450 nm.

### 2.6. Quantitative Gene Expression Evaluation

Synovial membrane samples were prepared for RNA extraction as described previously [12]. Samples were diluted to a concentration of 5 ng/*µ*L of RNA. The samples were assayed for quantitative gene expression levels in a qRT-PCR device (StepOnePlus Real-Time PCR System, Life Technologies, Carlsbad, CA, USA) using the SuperScript III platinum SYBR Green One-Step qRT-PCR kit (Life Technologies, Carlsbad, CA, USA). Primers were used for NF*κ*B, MMP-13, ADAMTS-4, COL2A1, COMP, and glyceraldehyde 3-phosphate dehydrogenase (GAPDH) (Table 1). These sequences were published previously [12]. The relative change in gene expression was determined via the comparative 2^−ΔΔCT^ method [21]. GAPDH was used as the internal control (housekeeping gene), and synovial membrane samples from all horses that were not incubated with any treatment were used as reference samples.

## 3. Statistical and Data Analysis

The statistical analysis was performed with SPSS 19.0 software (SPSS, IBM, NY, USA). The Shapiro-Wilk test was used to assess the fit of the data set to a normal distribution (goodness of fit). Data from hemoderivatives presented a normal distribution. However, data from SMEs presented a nonparametric distribution (*p* < 0.05). Several arithmetic transformations were performed to obtain normally distributed gene expression data from SMEs, but they were not successful to obtain this objective.

Platelet and WBC counts in whole blood and both PRP preparations were evaluated through a one-way analysis of variance (ANOVA), followed by Tukey's test. PDGF-BB and TGF-*β*_1_ from both PRG supernatants were compared using an unpaired *t*-test. Relative gene expression data were evaluated by a rank ANOVA (Kruskal-Wallis test), followed when necessary by the Mann–Whitney *U* test. A correlation analysis was performed to determine the Spearman correlation coefficient (*ρ*) between the relative expression of the genes in the study. A *p* value < 0.05 was accepted as statistically significant for all tests. Data are presented as either mean ± standard deviation (SD) or median (interquartile range (IR)).

## 4. Results

### 4.1. Cell and Growth Factor Concentration in Lc-PRP/Lc-PRG and Lr-PRP/Lr-PRG

Platelet counts were significantly (*p* < 0.05) different between whole blood, Lc-PRP, and Lr-PRP, with the lowest concentration for this last hemoderivative (97,600 ± 3,700 PLT/*µ*L), followed by whole blood (124,700 ± 3,800 PLT/*µ*L) and Lc-PRP (310,900 ± 20,500 PLT/*µ*L). WBC counts were also significantly different between the evaluated groups, with a higher concentration for Lc-PRP (36,900 ± 4,600 WBC/*µ*L), followed by whole blood (8,000 ± 4,700 WBC/*µ*L) and Lr-PRP (100 ± 30 WBC/*µ*L). The TGF-*β*_1_ concentration was similar between Lc-PRG (1766.3 ± 321.3 pg/mL) and Lr-PRG (1465.9 ± 19.8 pg/mL). PDGF-BB had a significantly (*p* < 0.05) higher concentration in Lc-PRG (3067.5 ± 946.8 pg/mL) when compared to Lr-PRG (378.6 ± 89.7 pg/mL).

### 4.2. Quantitative Gene Expression

**Figure 2:**
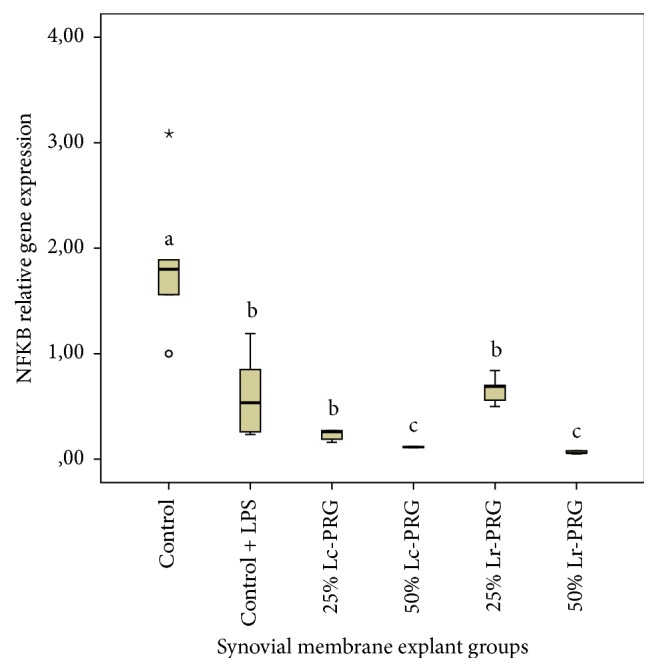
Box plot of median relative nuclear factor kappa B (NF*κ*B) gene expression. ^a–c^Lowercase letters denote significant differences (*p* > 0.05) between synovial membrane explant (SME) groups treated with leukoconcentrated platelet-rich gel (Lc-PRG) and leukoreduced platelet-rich gel (Lr-PRG) supernatants at different concentrations (25 and 50%). Groups with the same lowercase letter are not significantly different. ∘ denotes an outlier value, whereas *⋆* denotes extreme value; both symbols show nonparametric data.

NF*κ*B gene expression was significantly (*p* < 0.05) increased in SMEs from the control group in comparison with the SMEs of the control plus LPS group and those SME groups treated with both PRG supernatants at different concentrations (Figure 2). The expression of this gene was not significantly different between the SME control plus LPS group and the SME groups treated with both PRG supernatants at the concentration of 25%. Interestingly, the SME groups treated with both PRG supernatants at the concentration of 50% showed statistically significant lower (*p* < 0.05) NF*κ*B gene expression when compared with the other SME groups evaluated (Figure 2).

**Figure 3:**
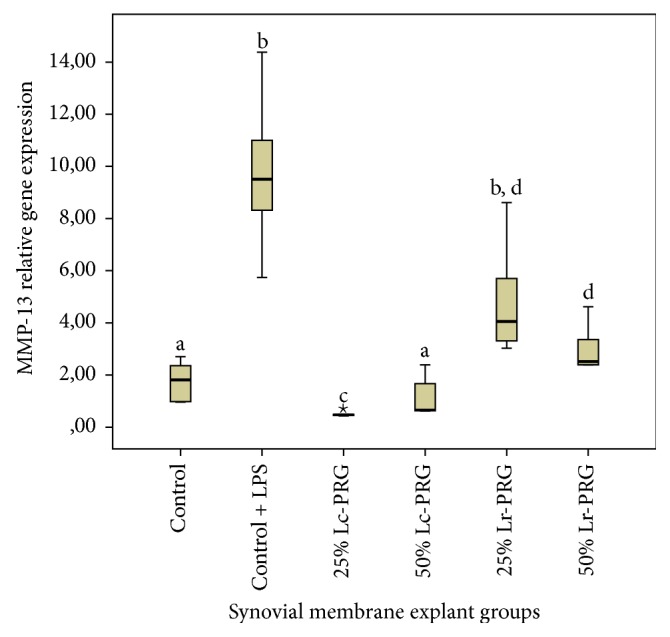
Box plot of median relative matrix metalloproteinase 13 (MMP-13) gene expression. ^a–d^Lowercase letters denote significant differences (*p* > 0.05) between synovial SME groups treated with Lc-PRG and Lr-PRG supernatants at different concentrations (25 and 50%). Groups with the same lowercase letter are not significantly different. *⋆* denotes extreme value, showing nonparametric data.

SMEs from the control group plus LPS presented significantly (*p* < 0.05) higher MMP-13 gene expression when compared to SMEs of the control group and those treated with the 25% and 50% Lc-PRG and 50% Lr-PRG supernatants (Figure 3). Notably, MMP-13 gene expression was significantly lower in SMEs cultured with the 25% Lc-PRG supernatant when compared to the remaining SME groups (Figure 3). In general, the expression of this gene was significantly (*p* < 0.05) lower in SMEs treated with Lc-PRG supernatants at both concentrations when compared with SMEs treated with Lr-PRG supernatants at the same concentrations.

**Figure 4:**
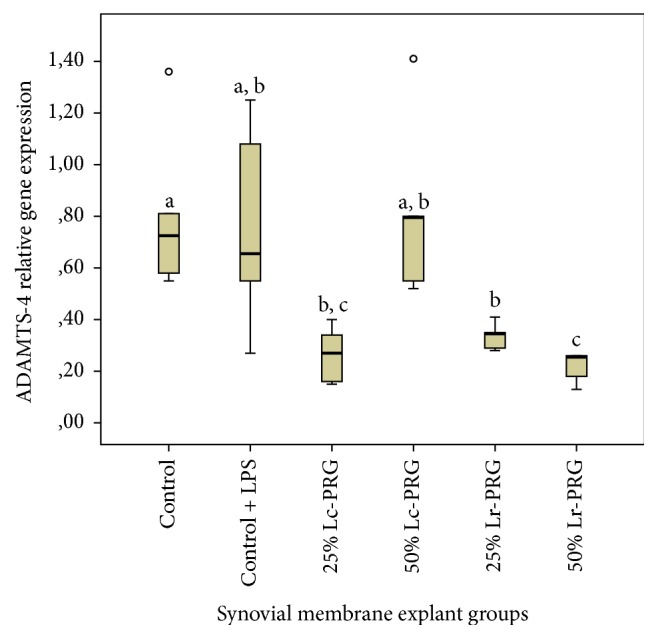
Box plot of median relative a disintegrin and metalloproteinase with thrombospondin motifs 4 (ADAMTS-4) gene expression. ^a–c^Lowercase letters denote significant differences (*p* > 0.05) between SME groups treated with Lc-PRG and Lr-PRG supernatants at different concentrations (25 and 50%). Groups with the same lowercase letter are not significantly different. ∘ denotes an outlier value, showing nonparametric data.

ADAMTS-4 gene expression was not significantly different between SMEs from the control group, the control group plus LPS, and the SMEs of the group treated with 50% Lc-PRG. The expression of this gene was also not significantly different between the SMEs of the control group plus LPS and those SMEs treated with both PRG supernatants at the concentration of 25% (Figure 4). Notably, the SMEs of the groups treated with 50% Lr-PRG supernatants showed significantly lower (*p* < 0.05) ADAMTS-4 gene expression than the remaining SME groups (Figure 4).

**Figure 5:**
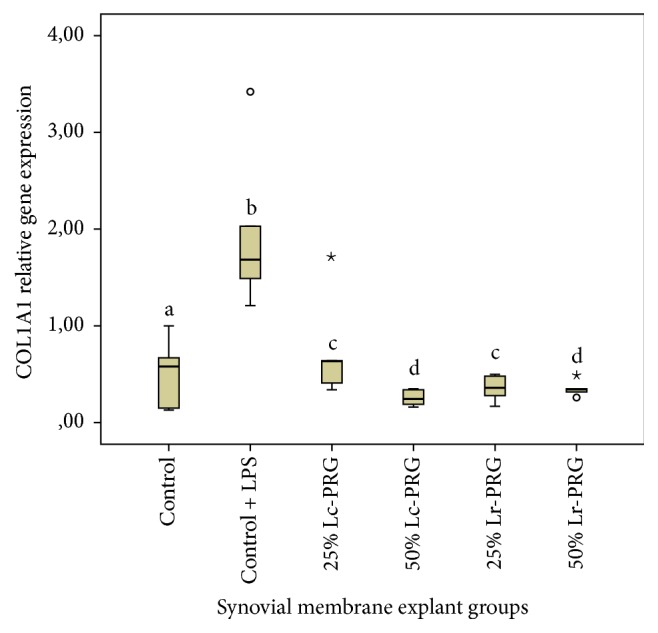
Box plot of median relative collagen type I alpha 1 (COL1A1) gene expression. ^a–d^Lowercase letters denote significant differences (*p* > 0.05) between SME groups treated with Lc-PRG and Lr-PRG supernatants at different concentrations (25 and 50%). Groups with the same lowercase letter are not significantly different. ∘ denotes an outlier value, whereas *⋆* denotes extreme value; both symbols show nonparametric data.

COL1A1 relative gene expression was significantly (*p* < 0.05) increased in SMEs in the control group challenged with LPS in comparison with the remaining SME groups (Figure 5). Notably, the expression of this gene was significantly (*p* < 0.05) lower in all SMEs treated with both PRG supernatants at both concentrations. However, both 50% PRG supernatants exhibited significantly lower (*p* < 0.05) COL1A1 relative gene expression than the homologous substances at a concentration of 50% (Figure 5).

**Figure 6:**
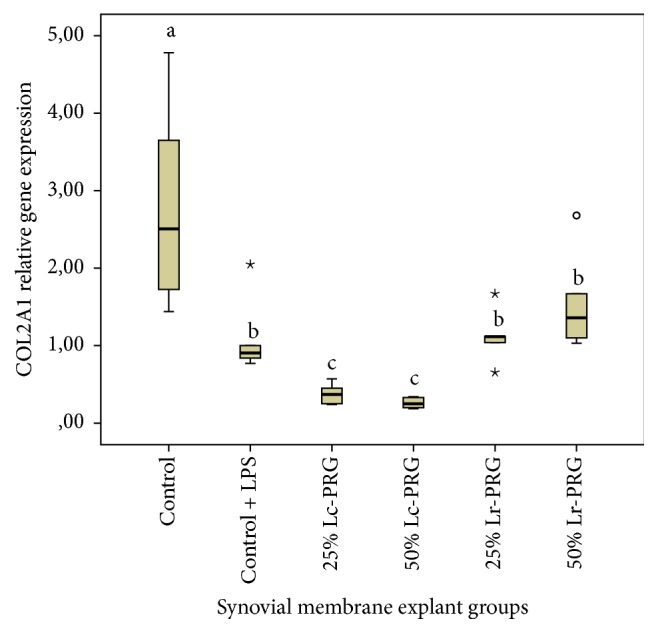
Box plot of median relative collagen type II alpha 1 (COL2A1) gene expression. ^a–c^Lowercase letters denote significant differences (*p* > 0.05) between SME groups treated with Lc-PRG and Lr-PRG supernatants at different concentrations (25 and 50%). Groups with the same lowercase letter are not significantly different. ∘ denotes an outlier value, whereas *⋆* denotes extreme value; both symbols show nonparametric data.

COL2A1 gene expression was significantly (*p* < 0.05) increased in SMEs from the control group in comparison to SMEs from the control group challenged with LPS and those SMEs cultured with both PRG supernatants at both concentrations (Figure 6). The expression of this gene was not significantly different between SMEs from the control group plus LPS and SMEs in the groups cultured with the Lr-PRG supernatants at both concentrations. Notably, SMEs cultured with Lc-PRG at both concentrations presented the most significantly reduced expression of this gene compared to the remaining SMEs groups (Figure 6).

**Figure 7:**
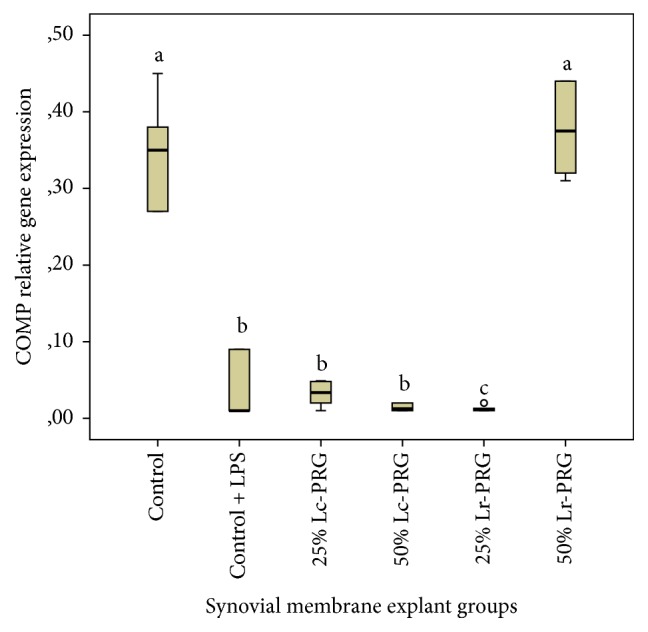
Box plot of median relative cartilage oligomeric matrix protein (COMP) gene expression. ^a–c^Lowercase letters denote significant differences (*p* > 0.05) between SME groups treated with Lc-PRG and Lr-PR supernatants at different concentrations (25 and 50%). Groups with the same lowercase letter are not significantly different. ∘ denotes an outlier value, showing nonparametric data.

COMP gene expression was significantly (*p* < 0.05) increased in SMEs from the control group and those cultured with 50% Lr-PRG supernatant in comparison with the SMEs of the remaining groups (Figure 7). Interestingly, COMP gene expression in SMEs of the control group plus LPS and SMEs of the groups cultured with Lc-PRG supernatants at both concentrations and the 25% Lr-PRG supernatant were not statistically different and remained upregulated in comparison to SMEs from the control group and those cultured with the 50% Lr-PRG supernatant (Figure 7).

A summary of the main effects affecting proinflammatory and anabolic gene expression in the SMEs is presented in Table 2.

### 4.3. Correlations

Significant negative correlations were observed between COLA1 and ADAMTS-4 gene expression (*ρ* = −0.60; *p* < 0.004), COMP and NF*κ*B gene expression (*ρ* = −0.63; *p* < 0.001), and COMP and ADAMTS-4 gene expression (*ρ* = −0.60; *p* < 0.003).

## 5. Discussion

The present study aimed to determine the relative expression (either down- or upregulation) of some proinflammatory (NF*κ*B, MMP-13, and ADAMTS-4) and anabolic (COL1A1, COL2A2, and COMP) genes implicated in OA pathophysiology in an in vitro system of equine synovitis, in which two different PRG supernatants at concentrations of 25% and 50% were evaluated. The results of this study partially indicate that inflamed synovial membrane responses to PRP preparations are different when compared to cartilage [12].

All PRG supernatants at different concentrations produced an interesting downregulation of proinflammatory genes (MMP-13 and ADAMTS-4) when compared to the gene expression observed in SMEs from the control group plus LPS. This anti-inflammatory effect was slight for the 25% Lr-PRG supernatant, moderate for the 25% Lc-PRG supernatant, and intense for the 50% Lc-PRG and 50% Lr-PRG supernatants. However, the Lc-PRG supernatants at both concentrations presented a slight to moderated catabolic effect, whereas the 25% Lr-PRG supernatant was slightly anabolic and the 50% Lr-PRG was intensely anabolic (Table 2).

We observed that NF*κ*B relative gene expression was downregulated in SMEs from the control group challenged with LPS. Interestingly, MMP-13 of the same SME group was upregulated, whereas ADAMTS-4 gene expression remained slightly increased without reaching an statistically significant level. Notably, the opposite NF*κ*B gene expression profile (upregulation) was observed in a similar in vitro study using cartilage explants [12]; however, in this last study, MMP-13 and ADAMTS-4 were also upregulated in cartilage explants challenged with LPS [12]. This finding could indicate that there is a differential response of NF*κ*B gene expression in equine cartilage and the synovial membrane when challenged with LPS and, MMP-13, and ADAMTS-4 upregulation in SMEs challenged with LPS could be related to an alternative way of inflammation not dependent on NF*κ*B gene expression. Notably, a similar molecular phenomenon has been described in synovial fibroblasts derived from rheumatoid arthritis patients in which MMP-13 upregulation was mediated by TGF-*β*_1_ and laminin and not via NF*κ*B upregulation [22]. However, it is also important to consider that perhaps some level of NF*κ*B activity could play an important role in synovial homeostasis and its complete suppression could be unfavorable for joint health. In this sense, NF*κ*B (at least in this in vitro system of synovitis) could be considered as a regulatory transcription factor and not as a proinflammatory gene.

COL1A1 gene expression was significantly upregulated in SMEs of the control group challenged with LPS, but all PRG supernatants at both concentrations downregulated the expression of this gene in the SME groups challenged with LPS such that expression was very similar to the expression of this gene in the healthy SME control group. Similar findings also were found in a similar in vitro study using equine cartilage explants [12]. This finding is important because PRP preparations may reverse the formation of fibrocartilage in patients with OA or better yet in cases of traumatic arthritis and avoid synovial membrane fibrosis.

On the other hand, COL2A2 and COMP gene expressions were drastically downregulated in SMEs challenged only with LPS. The upregulation of these genes was completely reversed only by the 50% Lr-PRG supernatant. This last finding was similar for equine cartilage explants challenged with LPS and cultured with the same substances at a similar concentration [12]. However, the 25% Lr-PRG supernatant led to better anabolic gene expression than the 50% Lr-PRG supernatant in that study [12].

Synovial membrane is a source of mesenchymal stem cells, which always is available to release cells with chondrogenic potential. Consequently, these cells could promote joint cartilage repair or regeneration in cases of OA or other inflammatory arthropathies [23]. It is plausible that COL2A1 gene expression in SMEs is related to the chondrogenic differentiation of stem cells contained in these tissues. Interestingly, we find that LPS produced an intense downregulation of this gene, which was significantly marked for SMEs cultured with both Lc-PRG supernatants. Notably, 50% Lr-PRG supernatant exhibited a trend to counteract the COL2A1 downregulation (catabolic) effect of LPS in this in vitro system of synovitis, which could indicate that PRP (particularly Lr-PRP) preparations could be useful to induce chondrogenic differentiation of stem cells from synovial membrane in patients with OA [24, 25].

The results of this study are complementary to a previous similar in vitro study [4] in which we measured, in the culture medium of LPS-challenged SMEs at 48 h and 96 h, the concentration of hyaluronan and some pro- (i.e., tumor necrosis factor alpha (TNF-*α*)) and anti-inflammatory (i.e., interleukin 4 (IL-4) and receptor antagonist of IL-1 (IL-1ra)) cytokines. In that study, the 25% Lc-PRG supernatant induced the most robust concentration of IL-1ra, whereas 50% Lr-PRG induced the most sustained production of IL-4 and hyaluronan [4]. These results, combined with those obtained in the present study, are useful to confirm that Lc-PRG supernatants at two concentrations can be classified as anti-inflammatory substances, possibly mediated by increased production of IL-1ra [4]; however, this anti-inflammatory mechanism may not be able to induce synovial membrane anabolism. On the other hand, the 50% Lr-PRG supernatant can be classified as an anti-inflammatory substance with intense anabolic effects, in which increased production of IL-4 could be involved [4]. It is known that this cytokine is able to induce cartilage ECM synthesis [26].

It is important to consider that although a healthy control group of SMEs was included, we decided to classify the therapeutic in vitro effect of the PRG supernatants as a function of their capacity to reverse the proinflammatory and catabolic effect of LPS on SMEs. Notably, none of the PRG supernatants (at both concentrations) evaluated in this study could produce a similar gene expression profile to what was observed in the healthy SMEs of the control group. At this point, the 50% Lr-PRG supernatant was able to induce the most similar gene expression profile when compared with the healthy SME group. These results have a very important biological value, because they demonstrate that Lr-PRG supernatants are able to counteract the proinflammatory and catabolic effects of LPS to some extent [8, 27].

Several correlations were observed in the present study. Notably, we observed that the expression of COMP and COL1A1 was negatively associated with the expression of NF*κ*B and ADAMTS-4. In general, the 50% Lr-PRG supernatant showed the most important anabolic effects, characterized by NF*κ*B and ADAMTS-4 downregulation and COMP upregulation. These findings are complementary to other PRP studies on joints [28, 29] and isolated cartilage [30] in which some researchers have concluded that Lr-PRP preparations are better to induce joint anabolism and to diminish inflammation than Lc-PRP preparations.

This study had several limitations, which should be addressed to avoid misinterpretation or exaggerated enthusiasm about the actual therapeutic potential of PRP in equine osteoarthritis or inflammatory (traumatic) arthritis. Autologous PRP preparations are frequently used for the treatment of equine patients with OA [31]. However, we used allogeneic PRP in the present study, which was obtained from blood of one donor mare. We processed the hemoderivatives (PRG supernatants) from the same animal in order to obtain substances with a standardized concentration of cells and proteins.

In general, the nature of this in vitro study was to evaluate the biological behavior of standard allogeneic hemoderivatives in the synovial tissues of genetically dissimilar horses to determine if these substances could affect the expression of selected genes implicated in OA. Moreover, this study could be useful to demonstrate that allogeneic PRP could be used for the treatment of OA horses. However, some of the beneficial effects observed in this system may have been influenced by immunological factors not associated with PRP.

It is known that one of the most important limitations for the dissemination of regenerative medicine products is the high costs related to the production of autologous products. Thus, there is constant interest in developing standardized and massive allogeneic biologic products free of pathogens and that do not induce immunologic rejections [32–34]. In line with this, it is necessary to evaluate the advantages and disadvantages of standardized allogeneic PRP preparations in treating musculoskeletal diseases in horses and humans [35].

## 6. Conclusion

This study demonstrates that both Lc- and Lr-PRP preparations at different concentrations can induce mixed anti-inflammatory and anabolic responses in an in vitro system of equine synovitis. The 25% and 50% Lc-PRG supernatants presented slight to moderate anti-inflammatory effects. However, these substances did not reverse the catabolic effects of LPS on SMEs, since they were unable to induce the upregulation of anabolic genes. On the other hand, 25% and 50% Lr-PRG supernatants showed intense anti-inflammatory and anabolic effects, which were greater for the 50% Lr-PRG supernatant. Additional in vitro studies in a coculture system of cartilage and synovial membrane explants and in patients with OA should be performed to assess the in vivo effect of PRP preparations at different concentrations.

## Figures and Tables

**Table 1:** Genes and primer sequences used in the study.

Targeted genes	Primer sequences (5′ → 3′)
GAPDH, glyceraldehyde-3-phosphate dehydrogenase 6	Forward TCCCTGCTTCTACTGGTGCT
	Reverse TGACAAAGTGGTCGTTGAGG
NF*κ*B, nuclear factor of kappa light polypeptide gene enhancer in B-cells-like 1	Forward CGATTTCGATATGGCTGTGA
	Reverse CACCTTCTTCAGCTCCTTGG
MMP 13, matrix metallopeptidase 13 (collagenase 3) 7	Forward GCATTCAAAAAGGCCTTCAA
	Reverse GGAAGCACAAAGTGGCTTTT
ADAMTS 4, metallopeptidase with thrombospondin type 1 motif 4	Forward TGTCAGCTTGGTGGTGACTC
	Reverse GTTGAAGACATGGCCCAGTT
COL1A1, collagen, type I, alpha 1 11	Forward AGCCAGCAGATCGAGAACAT
	Reverse CTGGCCACCATACTCGAACT
COL2A1, collagen, type II, alpha 1 6	Forward ACGTCCAGATGACCTTCCTG
	Reverse GTCCACACCAAATTCCTGCT
COMP, cartilage oligomeric matrix protein 21	Forward CCACGTGAATACGGTCACAG
	Reverse TAGGAACCAGCGGTAGGATG

**Table 2:** General proinflammatory and anabolic gene expression effects of several treatments applied to the synovial membrane explant (SME) groups in this study.^*∗*^

SME group	Relative gene expression in relation to GAPDH						Biological effect
	NFKB	MMP-13	ADAMTS-4	COL1A1	COL2A1	COMP	
Control group plus LPS	↑↑	↑↑↑	↑↑	↑↑	↑	↑	Proinflammatory and catabolic effect
25% Lc-PRG supernatant	↓	↓↓↓	↓↓	↓	↓↓	↓↓	Moderate anti-inflammatory but non anabolic effect
50% Lc-PRG supernatant	↓↓↓	↓↓	↓	↓↓	↓↓	↓↓	Intense anti-inflammatory but moderate catabolic effect
25% Lr-PRG supernatant	↓	↓	↓↓	↓	↑	↓↓↓	Slight anti-inflammatory and anabolic effect
50% Lr-PRG supernatant	↓↓↓	↓	↓↓↓	↓↓	↑↑	↑↑↑	Intense anti-inflammatory and intense anabolic effect

^*∗*^This classification was made only comparing the SME of the control group plus LPS and the SMEs groups cultured with both PRG supernatants at two concentrations plus LPS. ↑ = slight increase. ↑↑ = moderate increase. ↑↑↑ = intense increase. ↓ = slight decrease. ↓↓ = moderate decrease. ↓↓↓ = intense decrease.
